# Attention and cardiac phase boost judgments of trust

**DOI:** 10.1038/s41598-020-61062-7

**Published:** 2020-03-06

**Authors:** Xinyi Li, Michelle Chiu, Khena M. Swallow, Eve De Rosa, Adam K. Anderson

**Affiliations:** 1 Department of Human Ecology, Cornell University, Ithaca, NY USA; 2 Psychology Department, Temple University, Philadelphia, PA USA

**Keywords:** Neurophysiology, Human behaviour

## Abstract

Fluctuations in mental and bodily states have both been shown to be associated with negative affective experience. Here we examined how momentary fluctuations in attentional and cardiac states combine to regulate the perception of positive social value. Faces varying in trustworthiness were presented during a go/no-go letter target discrimination task synchronized with systolic or diastolic cardiac phase. Go trials lead to an attentional boosting of perceived trust on high trust and ambiguous neutral faces, suggesting attention both boosted existing and generated positive social value. Cardiac phase during face presentation interacted with attentional boosting of trust, enhancing high trust faces specifically during relaxed diastolic cardiac states. Confidence judgments revealed that attentional trust boosting, and its cardiac modulation, did not reflect altered perceptual or response fluency. These results provide evidence for how moment-to moment fluctuations in top-down mental and bottom-up bodily inputs combine to enhance *a priori* and generate *de novo* positive social value.

## Introduction

The coordinated integration of cognitive, affective and bodily processes is crucial to navigating the physical and social environment. This integration is demonstrated in how the perception of emotional value is modulated by the convergence of cognitive and bodily states, including central attention^1,2^ and peripheral physiological afferents^3–5^. There is now abundant evidence that affective value regulates attention^6–10^ as well as internal bodily states^11–13^. The specific directionality of these interactions has important implications for the mechanisms underlying emotional value. For instance, while attention interacts with objects with significant *a priori* value to increase their salience, it is now thought that attention itself can generate *de novo* affective value^3,5^, much like how W. James early theorizing suggested that bodily states have an intrinsic capacity to generate emotional feelings^14^. Here we considered how fluctuations in mental and bodily states contribute to the perception and generation of social value by examining how fluctuations in attentional and heart state are attributed to the trustworthiness of faces.

The act of ignoring certain stimuli in a target detection task decreases their emotional value relative to that of previously attended stimuli^15–17^. Relatedly, objects associated with a visual or auditory detection cue are later rated more valuable^3,18^. There remains some dispute around whether these results reflects valuation or devaluation^19^ and whether attention can generate *de novo* value, or is restricted to magnifying existing affective features, just as attention boosts stimulus perceptual features^18^. One approach to examining whether attention generates positive versus negative affect is to look to the body.

Elegant studies synchronizing stimulus presentation to cardiac phases have implicated brain regions such as the anterior cingulate, amygdala, insula, and brainstem nuclei that represent states of bodily arousal as well as modulate attentional and emotional processes^20–23^. Arterial baroreceptors fire strongly during systole, reflecting the transient increase in arterial blood pressure that follows heart contraction. Baroreceptor signals are transmitted to the brainstem via the vagus and glossopharyngeal nerves^24^, and then projected to forebrain regions^4,25^. It has been shown that cardiac systole improves the detection and intensity rating of threat related stimuli^21,26^, while under non-threat conditions relaxed parasympathetic diastolic heart states are associated with enhanced memory performance^20^ and decreased response inhibition^27^. Using cardiac gating, the synchronizing of stimulus processing with moment to moment fluctuating cardiac states, may provide an important window into how attention generates parasympathetic dominated positive or sympathetic dominant negative affect.

Bringing together mental and bodily states, attentional and cardiac modulation have been suggested to similarly depend on evolutionarily older noradrenergic nuclei originating from the brain stem locus coeruleus^4,28^. The potential underlying common origin of attentional and cardiac modulation of value suggests that momentary fluctuations in mental and bodily states interact to regulate our perception of value. Here we examined how phasic attention, via the attentional boost effect^29^ and cardiac phase^21^ come together to regulate perception of social value. Previous studies suggested that target detection requires more attention than distractor rejection^30–32^. Moreover, it has been shown that target detection enhances memory of temporally coincident, but task irrelevant background stimuli^29,33^. Research on this attentional boost effect demonstrates that the boost of encoding of targets is due to a target being rare and not a “go” response^34,35^. Thus, the attentional boost effect demonstrates that target detection of the foreground stimulus temporarily boosts the attention to the background stimulus, and provides a means to examine attentional enhancement of value. It has been proposed that enhanced gain and widespread cortical responses during target detection originates from the burst of activity of noradrenaline neurons in locus coeruleus (LC)^28^. To investigate how attention and cardiac phase jointly regulate perceived social value, we examined the evaluation of face value as indexed by the perception of trustworthiness^36,37^. Conceptually, facial trustworthiness is a measure of affiliation, is fundamental to social value^38^ and has been shown to well approximate general facial valence^39–41^. Physiologically, oxytocin has been shown to be associated with interpersonal trust^42^ and increased parasympathetic cardiac control^43^, which suggests cardiac phase may play a role in regulating trustworthiness. Methodologically, despite its subtle perceptual signal, studies have shown that people can form reliable trustworthiness judgements with minimal exposure to a face as short as 100 ms^44^, falling into the time scale of a heartbeat. In the realm of social value, if attention enhances value, this would not only increase perception of trustworthy faces, rather than modulate untrustworthy faces, but also be greater during diastole relative to systole.

Participants were presented with face stimuli overlaid with a letter in the center at each trial. Half the time the letter was target and required a response (Go trials). On the rest of the trials, the letter was a distractor that required no response (No Go trials). Attentional and cardiac gating of background visual stimulation were simultaneously manipulated using real-time ECG. Presentation of the face and letter was time locked to either cardiac systole or diastole. Participants were asked to rate the trustworthiness of each face immediately following the letter detection task. If attentional and cardiac gating regulate affect, then we expect the synchronization of attentional and cardiac states to interact to influence perceived trustworthiness. This would provide new evidence for how top-down mental and bottom-up bodily inputs come together to regulate our moment-to-moment sense of value.

## Methods

### Participants

35 participants (14 males, 21 females, age between 18 and 35) were recruited among students at Cornell University. Sample size was determined by a power analysis using GPower version 3.1.9.2^45^ based on previous studies on the main effects of attentional process^19,46^ and cardiac gating^21^ on emotional valence. Mean effect size (f2) in the studies on attentional process is 0.775. This determines a sample size of 18 to achieve a power of 85% with alpha = 0.05. The effect size (f^2^) of the study on cardiac gating is 0.40, and it determines a sample size of 32 to achieve a power of 85% with alpha = 0.05. All participants reported normal or corrected-to-normal vision and gave written informed consent prior to participation. All received course credit in exchange for their participation. All experimental protocols were approved by the Institutional Review Board of the Cornell University and procedures were carried out in accordance with the approved guidelines. Three participants were excluded due to error in heart recording. Another was excluded from data analysis due to chance level letter detection task performance (accuracy = 50.42%). This results in a final sample of N = 31 for further analysis.

### Stimuli

Stimuli consisted of 84 Caucasian faces randomly generated using FaceGen 3.1, obtained from the Social Perception Lab at Princeton University^36,37^. Each facial identity had 3 versions that varied on trust levels (−3, 0, 3 SD) based on the trustworthiness computer model generated by Oosterhof & Todorov^36^, where −3 SD was considered low trust, 0 was considered neutral, i.e., neither trustworthy or untrustworthy, and 3 SD high trust. Four distinct faces (each with 3 trustworthiness levels) were used in the practice trial. Another 80 distinct faces were used in the experimental task, totaling 240 faces (80 faces with 3 trustworthiness levels each).

### Experimental design

The task was programed by python 2.7 (http://www.python.org) using Pygame library 1.9.1 (http://www.pygame.org). Participants were provided with written instructions and a practice session consisting of 12 trials prior to the experimental task. The main task consisted of 240 trials divided into four blocks of 60 trials, where each trial consisted of a target detection task followed by an evaluation task. Between each experimental block, participants were given the option of a self-timed break. Using methodologies adapted from previous cardiac phase experiments^21,47^, the stimulus on each trial was time-locked to either cardiac systole or diastole. Trials were evenly divided according to factorial combination of letter condition (target or distractor), heart condition (systole or diastole) and trust levels (low, neutral, high) in each block.

### Heart phase recording

Electrocardiography (ECG) was acquired while participants sat in a chair, enabling assessment of heart peaks. Using the BioPac MP150, ECG signal was measured and transmitted at a rate of 2000 Hz via the Dual RSP/ECG BioNomadix amplifier system. Realtime detection of physiological heart peaks was achieved on a Windows computer through a scripted program in Python (https://github.com/rmarkello/rtpeaks), enabling the presentation of stimuli to be time-locked at systole or diastole. Stimuli were time locked to either the R wave, which is the end of diastole, or 300 ms after the R wave peak to synchronize with systole, accounting for the lag between baroreceptor activation and its communication to the brainstem and forebrain^23,48,49^.

### Target detection task

**Figure 1:**
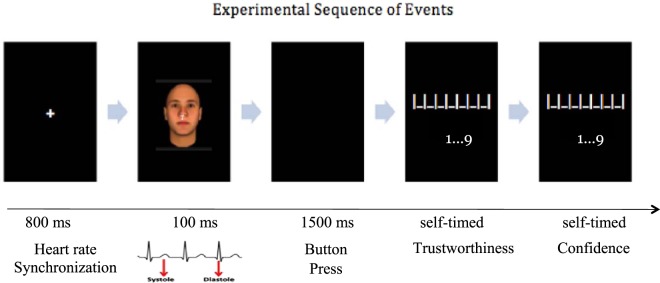
Experimental task and stimuli timing. Participants completed a target detection task where brief computer-generated faces were presented to coincide with systole and diastole phases of their cardiac cycle, followed by a trustworthiness-rating task. Each rating scale featured “very untrustworthy” or “very unconfident” below the lowest numeric value and “very trustworthy” or “very confident” below the highest numeric value. The face image was taken from Social Perception Lab database (http://tlab.princeton.edu/databases/) and has been reproduced with permission.

The letter detection task consisted of a fixation cross (800 ms + peak detection algorithm and cardiac phase lag), a combination stimulus consisting of a face with a letter in its center (100 ms), and a response window (1500 ms). Each participant was randomly assigned a target letter from a pool of five letters (X, H, T, L, or V), with the remaining four being distractors. Participants were instructed to press the space bar as quickly and accurately as they could during the response window only if the letter was a target letter (Fig. 1).

### Trustworthiness rating

Immediately, following target detection, on each trial, the rating task consisted of two self-timed screens that appeared one after the other. Participants were required to rate the immediately preceding face from the letter detection task on its trustworthiness and their confidence in their rating. Confidence was assessed as an index and control for how clearly participants saw the background face on each trial. Both ratings were on a 9-point scale ranging from 1 (‘very untrustworthy’ or ‘very unconfident’) to 9 (‘very trustworthy’ or ‘very confident’).

### Data analysis

Data analysis was performed in Python 3.4 and R 3.3.1 (https://www.r-project.org/). Real-time heart phase detection results were examined by a post-hoc peak detection algorithm. Trials where the time discrepancy between the actual and expected times of the letter and face display were larger than 50 ms were excluded.

### Statistical modeling

The influence of trust level (untrustworthy, neutral, trustworthy), cardiac phase (systole vs diastole), and attention (target/distractor letter) on trustworthiness ratings was assessed by a hierarchical linear mixed-effects regression model using lme4^50^ and emmeans (https://github.com/rvlenth/emmeans) packages in R. F values were computed using the joint_tests function from the emmeans package. By using random effects for subjects (n = 31) and distinct face stimuli (n = 80), we adjusted for the influence of different mean trustworthiness ratings associated with these variables. As fixed effects, we entered trust level, cardiac phase, and letter detection condition into the model. We also entered confidence rating as a continuous covariate to control for effects of attention on perceptual and response fluency.

## Results

### Letter detection

Accuracy among participants that met inclusion criteria was 96.72% ± 3.20% (mean ± standard error), indicating high performance overall. Neither letter detection accuracy nor reaction time (RT) significantly differed across systole and diastole trials (Paired t-test, accuracy: systole 96.53% ± 0.53%, diastole 96.91% ± 0.43%, t = 0.93, df = 3719, p = 0.35; RT: systole 486 ± 19 ms, diastole 488 ± 21 ms, t = 0.023, df = 925, p = 0.98), suggesting equivalent engagement of attention to the primary task across cardiac phase conditions.

### Cardiac phase detection

**Figure 2:**
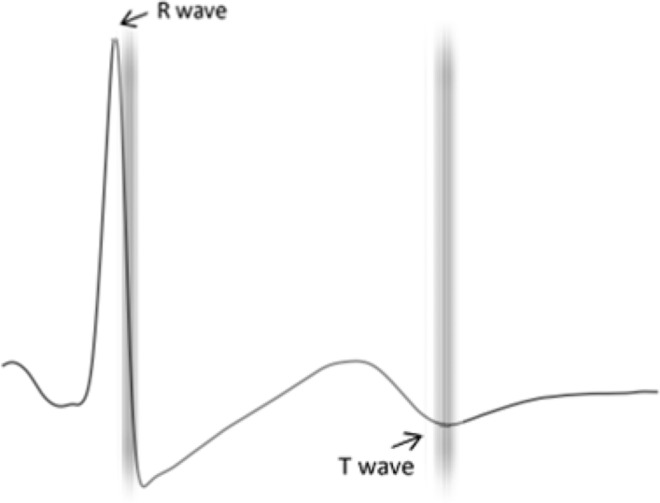
Stimulus presentation – heart phase alignment for trials included in further analysis. The labels depict R wave (cardiac diastole) and T wave (cardiac systole). The time delay between the two is 300 ms. For each trial across all participants, a vertical bar indicates when the face/letter stimulus was presented. Because most onsets were within 50 ms of the preceding dot, the lines are tightly clustered.

As a manipulation check we examined accuracy of stimulus synchronization with cardiac phase. Stimuli were well aligned to heart phase. Five trials (0.07%) in which the time discrepancy between stimuli onset and the actual heart phase was larger than 50 ms were excluded for further analysis across all participants. Figure 2 shows the alignment for the trials included in the analysis.

### Trustworthiness ratings regression model

Consistent with the face manipulation, there was a significant main effect of trust level (F = 2425.29, df = 7318.76, p < 10^−4^) on trustworthiness ratings. Pairwise comparison using emmeans confirmed that the average trustworthiness rating differs between every trust level pairs (β_high-low_ = 2.66 ± 0.038, t = 69.62, p < 10^−4^; β_medium-low_ = 1.41 ± 0.039, t = 36.53, p < 10^−4^; β_high-medium_ = 1.25 ± 0.038, t = 32.58, p < 10^−4^; collapsed across the other two conditions). The result shows that trustworthiness ratings progressively increased across trust levels, with neutral-trust face stimuli falling in between, rated neither trustworthy or untrustworthy, relative to the neutral middle point of the 9 pt scale (mean_rating_ = 4.45 ± 0.34).

**Figure 3:**
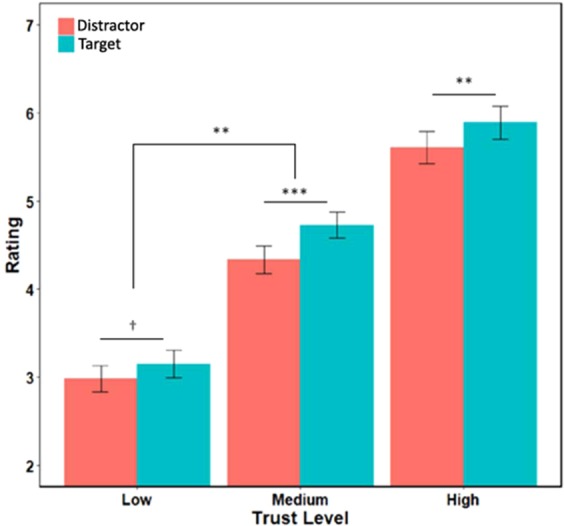
Main effect of target condition and two-way interaction. Faces paired with a target letter were in general rated as more trustworthy than faces paired with a distractor letter. This attentional boost of trust was largest in medium trust (neutral) faces, particularly relative to low trust faces. ^†^p < 0.1, *p < 0.05, **p < 0.01, ***p < 0.001.

Consistent with an attentional modulation of social value, there was a significant main effect of attention (β_target-distractor_ = 0.29 ± 0.08, F = 13.40, df = 76.03, p = 5 × 10^−4^) on trustworthiness ratings, in which faces presented with target letters were overall rated as more trustworthy (Mean = 4.59 ± 0.14) than those presented with distractor letters (Mean = 4.30 ± 0.14). There was also a significant two-way interaction between trust level and attention conditions (F = 4.57, df = 7316.13, p = 0.010) (Fig. 3). Attention modulated trust levels for high and neutral trust faces (β_high_ = 0.31 ± 0.091, t = 3.37, p = 0.001; β_neutral_ = 0.40 ± 0.091, t = 4.38, p < 10^−4^), with a nonsignificant trend for low trust faces (β_low_ = 0.17 ± 0.091, t = 1.87, p = 0.064). The effect of attention on trust ratings was significantly higher on neutral relative to low-trust faces (β_neutral-low_ = 0.23 ± 0.076, t = 3.00, p = 2.70 × 10^−3^), with a nonsignificant trend for high-trust greater than low-trust faces (β_high-low_ = 0.13 ± 0.076, t = 1.80, p = 0.072).

**Figure 4:**
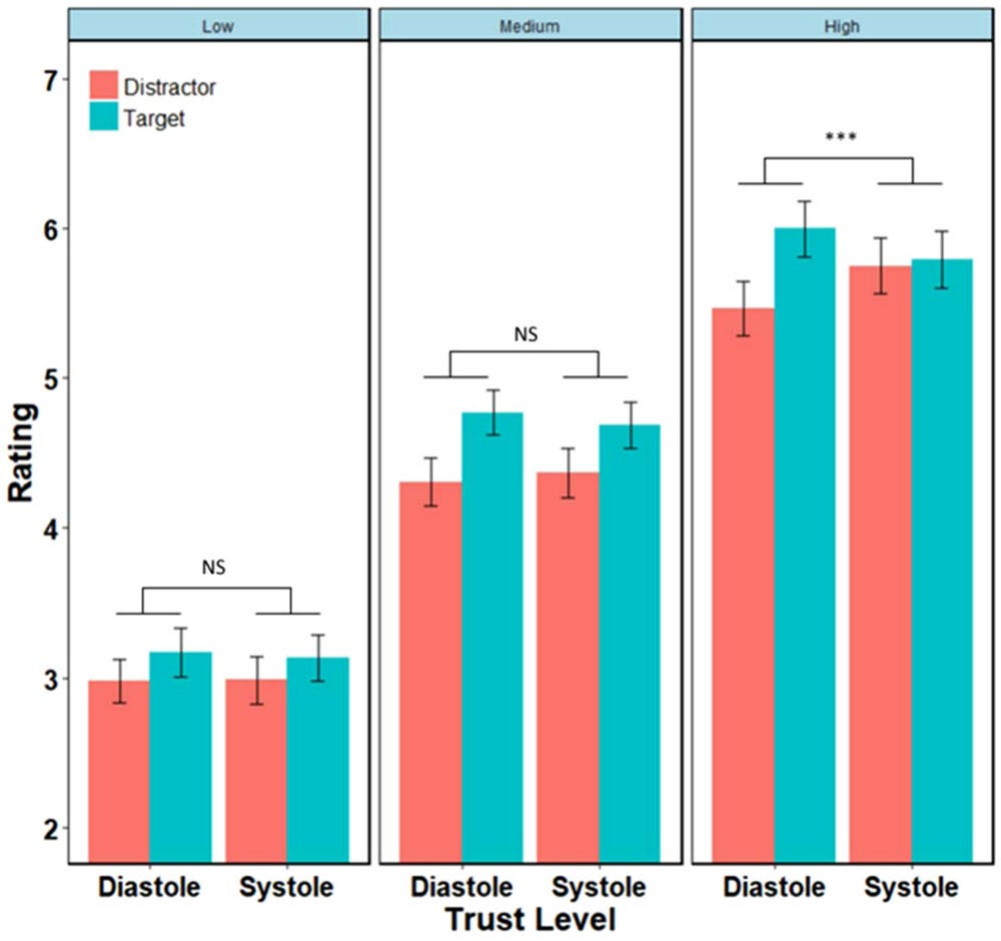
Effect of cardiac phase on trust ratings. Cardiac phase modulates the effect of attention on trustworthiness rating in high-trust faces, but not medium/neutral or low-trust faces.

The model also revealed a significant three-way interaction between attention condition, cardiac phase and trust level (F = 4.94, df = 7316.03, p = 7.2 × 10^−3^). Specifically, cardiac phase modulated the effect of attention on high-trust faces, supporting a two-way interaction between attention and heart phase (F = 7.46, df = 128.96, p = 7.2 × 10^−3^); whereby there was an increased attentional boost of trust ratings during diastole (β_target-distractor_ = 0.56 ± 0.13, t = 4.32, p < 10^−4^), but not systole (β_target-distractor_ = 0.06 ± 0.13, t = 0.45, p = 0.65, Fig. 4). Such an interaction was not present for neutral (F = 0.68, df = 128.89, p = 0.41) or low trust faces (F = 0.047, df = 128.89, p = 0.83).

Given the complexity of the design, the model was also run without the confidence rating as covariate to check for robustness. The results are qualitatively similar to the model including the covariate, and the results are reported in Supplementary Materials.

The model revealed a significant main effect of trust level (F = 2456.08, df = 7317.02, p < 10^−4^) on trustworthiness ratings. Pairwise comparison using emmeans confirmed that the average trustworthiness rating differs between every trust level pairs (β_high-low_ = 2.68 ± 0.038, t = 69.98, p < 10^−4^; β_medium-low_ = 1.47 ± 0.038, t = 38.26, p < 10^−4^; β_high-medium_ = 1.22 ± 0.038, t = 31.73, p < 10^−4^; collapsed across the other two conditions).

### Confidence ratings regression model

Although the above model included confidence as a covariate, we also submitted confidence ratings to a separate model. There was a significant main effect of trust level (F = 103.83, df = 7317.05, p < 0.001) on confidence ratings, with confidence rating greatest for lower trust faces and least for neutral (Mean_Low_ = 6.68 ± 0.20, Mean_medium_ = 6.18 ± 0.20, Mean_high_ = 6.49 ± 0.20, all pairwise comparison with p < 0.0001). The main effect of cardiac phase was not significant (F = 0.003, df = 76, p = 0.95). The model further revealed a main effect of attention condition (F = 6.04, df = 7317.05, p = 0.016). Pairwise comparison indicates higher confidence ratings in the target than distractor condition (β_target-distractor_ = 0.10 ± 0.04, t = 2.46, p = 0.016). There was also an interaction between attention and trust levels (F = 4.12, df = 7317.05, p = 0.016), where attention condition had a greater influence on confidence rating on high trust faces relative to lower trust faces and a nonsignificant trend relative to neutral faces (β_high-low_ = 0.20 ± 0.07, t = 2.84, p < 4.6 × 10^−3^, β_high-neutral_ = 0.13 ± 0.07, t = 1.80, p = 0.07).

## Discussion

In this study we investigated the role of mental. i.e., attention, and bodily feedback, i.e., cardiac gating, in boosting positive social value, specifically in the context of facial trustworthiness. Target but not distractor detection has been associated with an attentional boost to surrounding context in a similar go-no go paradigm^29^. We showed that such target detection enhances trustworthiness of not only trustworthy faces, but also for faces that were of neutral trust value, as well as a trend for untrustworthy faces. This is consistent with attention not only boosting value of faces that have trustworthy features but also adding value to neutral and untrusted ones. Attention did not render untrustworthy faces more untrustworthy but trended towards the opposite. This also indicates the effect of attention was to boost positive value rather than distractors enhancing negative value, as otherwise it would have been expected to demonstrate the biggest attentional effect for untrustworthy faces, the opposite result. We also demonstrated that cardiac phase modulated the effect of attention, primarily for high trust faces. Thus, peripheral bodily afferents by themselves did not add positive value, but rather enhanced the ability for attention to encode pre-existing value. While this interaction has been shown for threat signals^21^, here we show this extends to positive value, for faces judged highly trustworthy. This provides further evidence that in the present results attention increased positive value, as cardiac phase regulated the effect of attention on trustworthy but not untrustworthy faces. In sum the results are consistent with engendering *de novo* value in addition to boosting encoding of existing facial features diagnostic of trustworthiness.

Abundant studies have now investigated the effect of inattention and attention in regulating stimulus valence. Studies using the evaluated image stimuli itself as a target or distractor stimuli have shown a an inattention devaluation effect^46,51^, while studies using concurrent visual or auditory target/distractor cues, as done here, have shown, enhanced valuation of the image stimuli presented with Go cues^19,52^. These latter results echo findings in the visual memory domain. Faces presented with a superimposed target cue were later remembered better than faces presented with distractors, while memory for faces with distractors did not differ with faces presented alone, consistent with an “attentional boost”^33^. Due to the use of a Go/No-Go paradigm, these findings may be interpreted as due to an approach tendency. In contrast, research on the attentional boost effect demonstrates the boost of encoding of targets is due to a target being rare and not a “go” response. For instance, when no-go responses are rare, the encoding of stimuli associated with no-go trials are enhanced^35^. This boost effect also occurs with a silent counting and the magnitude of the effect is similar to that of a button press task^34^. Further, in contrast to a response inhibition account^46^, an attentional boost account for task irrelevant stimuli suggests that target stimuli induce a temporary spike in attentional resources which spills over to contemporaneous stimuli, resulting in an enhancement of later memory for background stimuli^28^. Most recentlySwallow & Atir (2018), using a similar paradigm as the current study showed that objects paired with a target cue were not only remembered better but were rated more highly than novel objects, although the memory enhancing and liking effects were independent of each other. Thus it is possible in our study that merely enhanced encoding fluency is associated with greater liking, as in the “mere exposure” effect^53^. By contrast, evidence from confidence ratings reveal greatest encoding confidence for low trust faces, where the effect of attention on positive value was least.

Does the perceived increase in trust rating merely reflects an attentional enhancement of the existing features in the stimuli, or an attentional generation of positive valence? If the former holds, we would expect to see high-trust faces paired with target letter rated more trustworthy, and low-trust faces rated even less trustworthy, resulting in a cross-over interaction. Instead, we observed comparable increase in trustworthiness ratings for both high- and low-trust faces, indicating attention modulates trustworthiness itself, instead of the perception of trustworthiness-related features. Previous studies on distractor devaluation also showed the effect regardless of stimuli’s valence^54^, indicating a general role for increased and decreased attention to alter subjective value.

With respect to a heart-brain connection, systolic cardiac phase has been shown to decrease memory performance, while increasing perception of threat^4,20^. Here we show cardiac phase modulates the attentional boost in trustworthiness ratings only for high trust faces. Further, targets significantly boosted the trustworthiness ratings of background faces when presented in diastole, but not in systole. In combination, these results are consistent with attention enhancing trust, increasing perception of positive social value. Cardiac phase did not show a direct influence on trustworthiness rating, but rather an attentional modulation of pre-existing trust features. This is consistent with previous findings that cardiac phase modulates attention to threat related stimuli^4^. Converging evidence suggests that threatening expressions draw more attention^55^, which may spare the need of additional attentional manipulation for cardiac phase to modulate these stimuli. Although varying in trust level, the low trust stimuli used in the current may not sufficiently signal threat and thus was not modulated by cardiac phase.

The attentional boost for background stimuli presented during target detection is proposed to reflect a phasic burst of noradrenaline in the locus coeruleus (LC) resulting in a brief burst of attentional resources^28,29^. Cardiac phase also influences LC activity, with systolic baroreceptor firing shown to inhibit noradrenaline neurons in the LC^56^ and decrease cortical noradrenaline^57^, making it plausible to attenuate the attentional boost. While recent results indicate some independence between cardiac gating and the attentional boost^58^, here we observed a significant attentional boost for high trust faces during diastole, but not systole. Visceral afferent signaling, which is known to be related with arousal, is often associated with emotionally salient events such as pain and fear^21,59^. Phasic responses of LC noradrenaline neurons have also been suggested to be elicited by salient events^60^. Consistent with this idea, LC noradrenaline activities have been shown to evoke basolateral amygdala neurons, and in turn promotes anxiety-like behavior^61^, which may alter the perception of fearful stimuli^21^. While untrustworthiness is associated with amygdala activity^62^, it is not known how this response interacts with cardiac phase. Further studies will need to investigate the neural underpinnings of how cardiac phase interacts with attention to regulate trustworthiness.

Together, our results showed that boosting attention generates positive social value. Cardiac cycle modulates this attention boosting effect, specifically for trustworthy faces during the relaxed diastolic cardiac phase where vagal parasympathetic afferents is highest. These findings have theoretical implications for understanding of how bodily and mental resources are integrated in the brain at the resolution of a heartbeat, supporting that social trust is regulated by fluctuations in states of the mind and heart.

## Supplementary information

Supplementary material.
