# Supplementary material for: Attention and cardiac phase boost judgments of trust
Source: Sci Rep. 2020 Mar 6;10:4274. doi: 10.1038/s41598-020-61062-7 (PMC7060330; doi:10.1038/s41598-020-61062-7)
Supplement: Supplementary file 1 — Supplementary material. [file 41598_2020_61062_MOESM1_ESM.docx]

**Attention and cardiac phase boost judgments of trust**

Xinyi Li^1*^, Michelle Chiu^2^, Khena M. Swallow^1^, Eve De Rosa^1^ & Adam K Anderson^1^

^1^Department of Human Ecology, Cornell University, Ithaca, NY

^2^Psychology Department, Temple University, Philadelphia, PA

*email: xl624@cornell.edu

Supplementary material

Mixed model without confidence rating as a covariate

To examine the robustness of the results of the regression model, we reran the regression model on the trustworthiness rating without confidence rating. The model is the same as the one presented in the main text, except the confidence ratings were not entered as a covariate. The results are qualitatively the same as the original model with the covariate.

There was a significant main effect of attention (β_target-distractor_ = 0.28 ± 0.08, F = 12.00, df = 76.00, p = 9 x 10^-4^) on trustworthiness ratings, in which faces presented with target letters were overall rated as more trustworthy (Mean = 4.59 ± 0.14) than those presented with distractor letters (Mean = 4.31 ± 0.14). There was also a significant two-way interaction between trust level and attention conditions (F = 4.18, df = 7317.02, p = 0.015). Attention modulated trust levels for high and neutral trust faces (β_high_ = 0.29 ± 0.093, t = 3.08, p = 2.5 x 10^-3^; β_neutral_ = 0.39 ± 0.093, t = 4.22, p < 10^-4^), with a nonsignificant trend for low trust faces (β_low_ = 0.17 ± 0.093, t = 1.87, p = 0.069). The effect of attention on trust ratings was significantly higher on neutral relative to low-trust faces (β_neutral-low_ = 0.22 ± 0.077, t = 2.89, p = 3.9 x 10^-3^), and the difference between high-trust greater than low-trust faces is not significant (β_high-low_ = 0.12 ± 0.077, t = 1.51, p = 0.13).

The model also revealed a significant three-way interaction between attention condition, cardiac phase and trust level (F = 4.88, df = 7317.02, p = 7.7 x 10^-3^). Specifically, cardiac phase modulated the effect of attention on high-trust faces, supporting a two-way interaction between attention and heart phase (F = 7.25, df = 127.41, p = 8.0 x 10^-3^); whereby there was an increased attentional boost of trust ratings during diastole (β_target-distractor_ = 0.54 ± 0.13, t = 4.08, p < 10^-4^), but not systole (β_target-distractor_ = 0.04 ± 0.13, t = 0.27, p = 0.78, Figure 4). Such an interaction was not present for neutral (F = 0.60, df = 127.33, p = 0.44) or low trust faces (F = 0.057, df = 127.33, p = 0.81).
